# Predicting protein-binding regions in RNA using nucleotide profiles and compositions

**DOI:** 10.1186/s12918-017-0386-4

**Published:** 2017-03-14

**Authors:** Daesik Choi, Byungkyu Park, Hanju Chae, Wook Lee, Kyungsook Han

**Affiliations:** Department of Computer Science and Engineering, Inha University, Incheon, 22212 South Korea

**Keywords:** Protein-binding region, RNA-protein interaction, Prediction method

## Abstract

**Background:**

Motivated by the increased amount of data on protein-RNA interactions and the availability of complete genome sequences of several organisms, many computational methods have been proposed to predict binding sites in protein-RNA interactions. However, most computational methods are limited to finding RNA-binding sites in proteins instead of protein-binding sites in RNAs. Predicting protein-binding sites in RNA is more challenging than predicting RNA-binding sites in proteins. Recent computational methods for finding protein-binding sites in RNAs have several drawbacks for practical use.

**Results:**

We developed a new support vector machine (SVM) model for predicting protein-binding regions in mRNA sequences. The model uses sequence profiles constructed from log-odds scores of mono- and di-nucleotides and nucleotide compositions. The model was evaluated by standard 10-fold cross validation, leave-one-protein-out (LOPO) cross validation and independent testing. Since actual mRNA sequences have more non-binding regions than protein-binding regions, we tested the model on several datasets with different ratios of protein-binding regions to non-binding regions. The best performance of the model was obtained in a balanced dataset of positive and negative instances. 10-fold cross validation with a balanced dataset achieved a sensitivity of 91.6%, a specificity of 92.4%, an accuracy of 92.0%, a positive predictive value (PPV) of 91.7%, a negative predictive value (NPV) of 92.3% and a Matthews correlation coefficient (MCC) of 0.840. LOPO cross validation showed a lower performance than the 10-fold cross validation, but the performance remains high (87.6% accuracy and 0.752 MCC). In testing the model on independent datasets, it achieved an accuracy of 82.2% and an MCC of 0.656. Testing of our model and other state-of-the-art methods on a same dataset showed that our model is better than the others.

**Conclusions:**

Sequence profiles of log-odds scores of mono- and di-nucleotides were much more powerful features than nucleotide compositions in finding protein-binding regions in RNA sequences. But, a slight performance gain was obtained when using the sequence profiles along with nucleotide compositions. These are preliminary results of ongoing research, but demonstrate the potential of our approach as a powerful predictor of protein-binding regions in RNA. The program and supporting data are available at http://bclab.inha.ac.kr/RBPbinding.

**Electronic supplementary material:**

The online version of this article (doi:10.1186/s12918-017-0386-4) contains supplementary material, which is available to authorized users.

## Background

Interactions between protein and RNA molecules are essential to various cellular processes, such as post transcriptional gene regulation, translation, and alternative splicing [1]. Many studies have been conducted to identify RNA-binding proteins (RBPs) or binding sites in protein and RNA molecules. In particular, recent advances in high-throughput experimental technologies, including next-generation sequencing technologies and cross-linking and immunoprecipitation (CLIP), have accelerated the discovery of RBPs and their target RNAs. Despite the increased number of known RBPs and their target RNAs, the mechanism of protein-RNA interactions is not fully uncovered and a large number of RBPs and their target RNAs remain to be uncovered. For example, for the ∼20,500 protein-coding genes in humans, only 1,542 RBPs (7.5%) and their target RNAs have been identified so far [2].

As a complement to experimental methods, several computational methods have been proposed, which are largely motivated by the increased amount of data on protein-RNA interactions and the availability of complete genome sequences of several organisms. Computational methods in general are much less time-consuming and costly than experimental methods.

Most existing computational methods are primarily limited to finding RNA-binding sites in proteins instead of protein-binding sites in RNAs. For instance, BindN+ [3], an upgraded version of BindN [4], uses a support vector machine (SVM) to predict the RNA- or DNA-binding residues from biochemical features and evolutionary information of protein sequences. RNABindRPlus [5] also predicts RNA-binding residues in a protein sequence by combining predictions from an optimized SVM and those from a sequence homology method. aaRNA [6] predicts RNA binding residues in protein using sequence- and structure-based features.

Compared to the task of predicting RNA-binding sites in proteins, predicting protein-binding sites in RNA is more challenging for several reasons [7]. Until very recently, there were few computational methods that can predict protein-binding sites in RNA. catRAPID estimates the binding propensity of RNA and protein molecules by combining secondary structure, hydrogen bonding and van der Waals contributions [8]. It often predicts an entire RNA sequence as a binding site even for an RNA sequence of 50 or more nucleotides. DeepBind [9] is known to outperform state-of-the-art experimental and computational methods. It uses deep convolutional neural networks, trained on a huge amount of data from high-throughput experiments. For the problem of predicting RBP-binding sites in RNA sequences, DeepBind was trained on data from RNAcompete, CLIP-seq and RIP-seq [10]. It contains ∼200 distinct models, each for different RBPs, so the user should try all of them in the absence of prior information on RBP. As output, it only provides a predictive binding score without protein-binding sites in the input RNA sequence. A new prediction model called PRIdictor [11, 12] predicts binding sites in RNA and protein sequences at the nucleotide and residue level. Wong et al. [13] developed a method that predicts interacting nucleotides and residues between DNA and proteins.

In this paper, we propose a new method for predicting protein-binding regions in mRNA, which are associated with post-transcriptional regulation of gene expression. The method uses sequence profiles constructed from log-odds scores of mono- and di-nucleotides and sequence compositions of mono-, di- and tri-nucleotides. As shown in the paper, the proposed method showed a high performance in testing on a large number of human RNA sequences and was substantially better than other methods. The rest of the paper presents the details of our approach and its experimental results.

## Methods

### Datasets

We obtained protein-binding sites in RNAs from CLIPdb [14], which provides curated published CLIP-seq data sets for four species (human, mouse, worm, and yeast). To obtain a sufficient amount of reliable data, we restricted the data to those binding regions of 25 nucleotides in ‘+’ strands of human mRNAs, which were identified by PAR-CLIP technology [15] and have the binding affinity score > 0.9 in PARalyzer [16]. Human mRNAs were selected against others because the largest amount of RBP binding sites is known in human mRNAs. Different RBPs are known to have different binding preferences within an mRNA. We examined the type of RBP binding regions in the extracted human mRNAs by mapping the Ensembl transcripts to the GRCh37 assembly. Coding sequence (CDS) regions of mRNA are the most frequent binding regions of RBPs, followed by 3^′^ UTR (Additional file 1).

The reason for selecting 25 nucleotides as the size of a binding region is because protein-binding regions identified by PAR-CLIP are typically between 21 and 35 nucleotides in length, and binding regions of 25 nucleotides resulted in the larger amount of data from CLIPdb than other choices for the size (see Additional file 2 for the distribution of the length of RBP-binding regions). After extracting a total of 5,145 RBP-binding regions for 14 RBPs, we assembled RNA sequences using the reference human genome GRCh37/hg19. These RNA sequences were used as positive data in our study (Additional file 3). RBP sequences were obtained from NCBI GEO (http://www.ncbi.nlm.nih.gov/geo/).

For negative data, we selected 51,450 (10-fold of the positive data) non-binding regions of 25 nucleotides in the same reference human genome GRCh37/hg19. The human genome contains more non-binding regions than protein-binding regions, so we constructed several datasets with different ratios of binding to non-binding regions (called 1:1, 1:2, 1:4, 1:6, 1:8 and 1:10 datasets hereafter).

In order to remove redundancy in the datasets, we first executed CD-HIT-EST [17] on each of the six datasets (1:1, 1:2, 1:4, 1:6, 1:8 and 1:10 datasets) and removed those with a sequence similarity of 80% or higher. After removing similar sequences, 4372 sequences out of the 5,145 RBP-binding sequences were left. The remaining 4372 RBP-binding sequences were partitioned into two datasets: training dataset (70% of the remaining RBP-binding sequences) and test dataset (30%). Thus, there are no similar RNA sequences between training and test datasets and within training or test datasets. Table 1 shows the number of sequences in the training and test datasets with different ratios of positive to negative instances. Since the redundancy removal was enforced separately in the 1:1, 1:2, 1:4, 1:6, 1:8 and 1:10 datasets, the ratio of positive to negative instances may not be exactly 1:*n* (*n*=1,2,4,6,8,10) (see Additional files 4 and 5).

**Table 1:** Number of RNA sequences in training and test datasets

P:N	1:1	1:2	1:4	1:6	1:8	1:10
Training						
Dataset	3,372:3,679	3,372:7,200	3,372:13,611	3,372:19,065	3,372:22,826	3,372:26,212
Subtotal	7,051	10,572	16,983	22,473	26,198	29,584
Test						
Dataset	1,000:1,000	1,000:2,000	1,000:3,998	1,000:5,998	1,000:7,998	1,000:9,998
Subtotal	2,000	3,000	4,998	6,998	8,998	10,998
Total	9,051	13,572	21,981	29,435	35,196	40,582

Since similar sequences were removed separately in each 1:*n* dataset, the number of negative data (N) is not an exact multiple of the number of positive data (P)

### Nucleotide profiles and compositions

We constructed position weight matrices (PWMs) of two types: (1) mono-nucleotide position weight matrix (mPWM) and (2) di-nucleotide position weight matrix (dPWM). mPWM(*i,j*) represents the log-odds score of the *i*-th nucleotide (*i*=1,2,3,4) in the *j*-th position (*j*=1,2,…, sequence length *n*), which is defined by Eq. 1. Likewise, dPWM(*d* *i,j*) represents the log-odds score of the *di*-th di-nucleotide (*di*=1,2,…,16) in the *j*-th position (*j*=1,2,…, *n*−1), defined by Eq. 2.

1 $$\begin{array}{@{}rcl@{}} mPWM(i, j) &=& \ln \left(\frac{frequency^{+}(i, j)}{frequency^{-}(i, j)}\right) \end{array} $$

2 $$\begin{array}{@{}rcl@{}} dPWM(di, j) &=& \ln \left(\frac{frequency^{+}(di, j)}{frequency^{-}(di, j)}\right) \end{array} $$

**Fig. 1:**
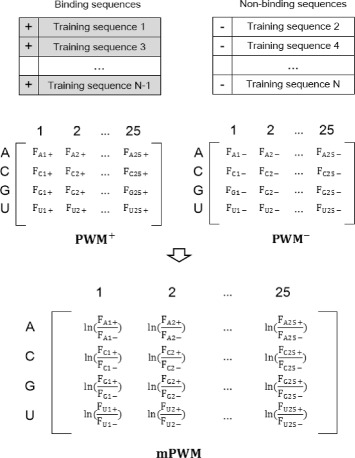
Construction of mono-nucleotide position weight matrix (mPWM). Both binding and non-binding sequences are used to generate an mPWM, in which each element (*i,j*) represents the log-odds score of the *i*-th nucleotide (*i*=A, C, G and U) in the *j*-th position (*j*=1,2,…, sequence length *n*). *F* in PWM ^+^, PWM ^−^ and mPWM denotes the frequency of a nucleotide at a position

The PWM of mono-nucleotides, also known as position specific score matrix (PSSM) or sequence profile, is frequently used with slightly different definitions [3, 18]. We computed PWM ^+^ and PWM ^−^ from a training dataset of protein-binding sequences and non-binding sequences, respectively (see Fig. 1). Each element of PWM ^+^ and PWM ^−^ represents the frequency of *i*-th nucleotide (*i* is any one of A, C, G and U) in the *j*-th position of RNA of *n* nucleotides. We combined PWM ^+^ and PWM ^−^ of a training dataset into mPWM by Eq. 1, which represents the log-odds score the *i*-th nucleotide in the *j*-th position.

The PWM of di-nucleotides (dPWM) is less commonly used than PWM of mono-nucleotides, but can elucidate higher order structures of protein-binding sequences. We built dPWM in a similar way to mPWM. We first constructed dPWM ^+^ and dPWM ^−^ from a training dataset of protein-binding sequences and non-binding sequences, respectively. Each element of dPWM ^+^ and dPWM ^−^ represents the frequency of the *di*-th di-nucleotide (*di* is any one of AA, AC, …, UU) in the *j*-th position (*j*=1,2,…, *n*−1) of RNA of *n* nucleotides. dPWM ^+^ and dPWM ^−^ of a training dataset were combined into dPWM, which represents log-odds score the *di*-th di-nucleotide in the *j*-th position. The same mPWM and dPWM generated from a training dataset were used in both training and testing the prediction model.

**Fig. 2:**
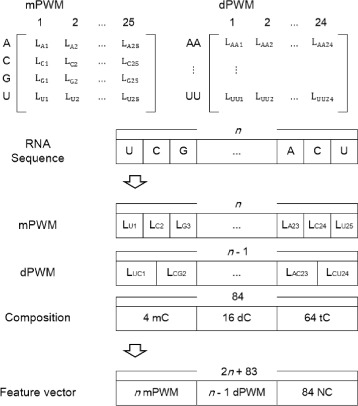
Structure of a feature vector. For a sequence of *n* nucleotides, mPWM and dPWM are represented by *n* and *n*−1 elements, respectively. Compositions represent the frequency of each mono-nucleotide (4 elements), di-nucleotide (16 elements) and tri-nucleotide (64 elements) in the RNA sequence. A protein sequence is represented by 63 elements (7 compositions, 21 transitions and 35 distributions)

In addition to the position weight matrices of two types, we computed nucleotide compositions of three types: mono-nucleotide composition (mC), di-nucleotide composition (dC) and tri-nucleotide composition (tC). Thus, a single RNA sequence of *n* nucleotides is represented in a feature vector with 2*n*+83 elements (*n* elements for mPWM, *n*−1 elements for dPWM, and 84 elements for nucleotide compositions). For a sequence of 25 nucleotides, a single feature vector contains 133 elements (see Fig. 2 for the structure of a feature vector).

### Protein features

To represent a protein sequence, 20 amino acids are first clustered into 7 groups {A, G, V}, {C}, {M, S, T, Y}, {F, I, L, P}, {H, N, Q, W}, {K, R} and {D, E} based on their dipoles and volumes [19]. Every amino acid in each protein sequence is transformed into an index representing an amino acid group. For each protein sequence, the composition, transition, and distribution of amino acid groups are represented in a feature vector [19]. The composition is the normalized frequency of each group in the protein sequence. The transition is the normalized frequency of transition between each group in the protein sequence. The distribution is the normalized position of the first, 25, 50, 75 and 100%-th amino acid of each group in the protein sequence. A protein sequence is represented by a feature vector with 63 elements (7 compositions, 21 transitions, and 35 distributions). Thus, a model that predicts RBP binding sites using both RNA and proteins features require 63 more elements in a feature vector than that using RNA features only.

### Prediction model

We built a support vector machine (SVM) model using a library for support vector machine (LIBSVM) [20]. As a kernel the radial basis function (RBF) was selected instead of the linear kernel because the number of instances (> 100,000 RNA sequences) in our dataset is much larger than the number of features (≈ 200). Besides, it is known that there is no need to consider linear SVM if complete model selection has been conducted using the Gaussian kernel [21].

The SVM model with the RBF kernel has two parameters, cost (C) and *γ*. We determined the best parameter values (C = 32 and *γ*=0.0078125) by running the grid search tool of LIBSVM on the training dataset. Unless specified otherwise, all the results shown in this paper were obtained with C = 32 and *γ*=0.0078125.

For comparative purposes, we also built another model using WEKA random forest (http://www.cs.waikato.ac.nz/ml/weka/). As discussed later in the Results and discussion section, the SVM model was chosen as the final model for the web server after it was compared with the random forest model. The results of the random forest model shown in this paper were obtained with 60 trees and 25 features, which resulted in the best performance.

### Evaluation of the model

The performance of the SVM and random forest models was evaluated using six measures: sensitivity, specificity, accuracy, positive predictive value (PPV), negative predictive value (NPV), and Matthews correlation coefficient (MCC), which are defined as follows.

3 $$ Sensitivity = \frac{TP}{TP+FN}  $$

4 $$ Specificity = \frac{TN}{TN+FP}  $$

5 $$ Accuracy = \frac{TP+TN}{TP+FP+TN+FN}  $$

6 $$ PPV = \frac{TP}{TP+FP}  $$

7 $$ NPV = \frac{TN}{TN+FN}  $$

8 $$ {} MCC = \frac{(TP\times TN)-(FP\times FN)}{\sqrt{(TP+FP)(TP+FN)(TN+FP)(TN+FN)}}  $$

True positives (TP), true negatives (TN), false positives (FP), and false negative (FN) represent correctly predicted binding regions, correctly predicted non-binding regions, non-binding regions that are incorrectly predicted as binding, and binding regions that are incorrectly predicted as non-binding, respectively.

As described above, our prediction model uses PWM of two types and nucleotide compositions as RNA features. To examine the contribution of the features to the prediction performance, we tried different combinations of features in 10-fold cross validation.

We evaluated the model in several different ways. First, we performed two types of cross validation: (1) standard 10-fold cross validation with six different training datasets (1:1, 1:2, 1:4, 1:6, 1:8 and 1:10 training datasets) and (2) leave-one-protein-out (LOPO) cross validation [22] with the 1:1 training dataset. The reason for performing LOPO cross validation is because typical *k*-fold cross validation tends to over-estimate predictive performances for paired inputs such as protein-protein interactions (PPIs) or protein-RNA interactions. Recently Park and Marcottee [23] and Hamp and Rost [24] have demonstrated that both standard and refined cross validations lead to inflated accuracy of PPI prediction methods. In LOPO cross validation with respect to RBPs, all RNA sequences (both RBP-binding and non-binding sequences) for one RBP are taken out for testing and remaining RNA sequences are used for training.

In addition to cross validations of two types, we also tested the SVM model on independent datasets, which were not used in training the model. We also compared our SVM model with DeepBind [9] and catRAPID [8] using another test dataset. Out of the 14 RBPs used in our study, DeepBind provides 7 distinct models, one for each of 7 RBPs (FUS, FXR1, FXR2, IGF2BP2, LIN28A, QKI, TARDBP). For a fair comparison, we extracted new 700 RBP-binding regions of 25 nucleotides from CLIPdb (100 RBP-binding regions for each of the 7 RBPs). To remove redundancy between the 700 RNA sequences and the training dataset, we executed CD-HIT-EST-2D on them with a cut-off value of 0.8. (see Table 2 for the number of remaining RNA sequences after running CD-HIT-EST-2D).

**Table 2:** Results of testing our model and DeepBind on RNA sequences of 25 nucleotides. catRAPID could not be tested on RNA sequences of 25 nucleotides since the minimum length of an RNA sequence required by catRAPID is 50 nucleotides

	#RBP-binding						
RBP	RNA regions	Sensitivity	Specificity	Accuracy	PPV	NPV	MCC
Our model							
FUS	64	93.75%	94.00%	93.90%	90.91%	95.92%	0.873
FXR1	67	97.01%	94.00%	95.21%	91.55%	97.92%	0.902
FXR2	80	66.25%	94.00%	81.67%	89.83%	77.69%	0.638
IGF2BP2	79	74.68%	94.00%	85.47%	90.77%	82.46%	0.709
LIN28A	82	85.37%	94.00%	90.11%	92.11%	88.68%	0.801
QKI	77	84.42%	94.00%	89.83%	91.55%	88.68%	0.793
TARDBP	94	12.77%	94.00%	54.64%	66.67%	53.41%	0.117
Weighted average		**70.72%**	**94.00%**	**83.83%**	**90.14%**	**80.54%**	**0.676**
DeepBind							
FUS	64	32.81%	42.00%	38.41%	26.58%	49.41%	-0.246
FXR1	67	11.94%	44.00%	31.14%	12.50%	42.72%	-0.444
FXR2	80	15.00%	55.00%	37.22%	21.05%	44.72%	-0.320
IGF2BP2	79	41.77%	51.00%	46.93%	40.24%	42.58%	-0.072
LIN28A	82	12.20%	52.00%	34.07%	17.24%	41.94%	-0.382
QKI	77	83.12%	75.00%	78.53%	71.91%	85.23%	0.576
TARDBP	94	52.13%	92.00%	72.68%	85.96%	67.15%	0.484
Weighted average		**36.28%**	**58.71%**	**48.91%**	**40.53%**	**54.29%**	**–0.051**

The specificity of our method is the same for all RBPs because it used a same set of negative data for all RBPs with a single model, whereas DeepBind has distinct models for each RBP

Since catRAPID requires an RNA sequence of at least 50 nucleotides, we extended the RBP-binding regions by including 13 nucleotides on each side of the binding regions in their original genome sequences. Redundancy between the extended RNA sequences and the training dataset was removed by running CD-HIT-EST-2D on them with a cut-off value of 0.9 because instead of 0.8 since the cut-off value of 0.8 removed too many RNA sequences (see Table 3 for the number of remaining RNA sequences after running CD-HIT-EST-2D). As negative data for the 700 RNA sequences, we extracted additional 100 non-binding regions of 25 and 51 nucleotides in the reference human genome GRCh37/hg19.

**Table 3:** Results of testing our model, DeepBind and catRAPID on RNA sequences of 51 nucleotides

	#RBP-binding						
RBP	RNA regions	Sensitivity	Specificity	Accuracy	PPV	NPV	MCC
Our model							
FUS	100	79.00%	70.00%	74.50%	72.48%	76.92%	0.492
FXR1	97	88.66%	70.00%	79.19%	74.14%	86.42%	0.596
FXR2	93	69.89%	70.00%	69.95%	68.42%	71.43%	0.399
IGF2BP2	94	55.32%	70.00%	62.89%	63.41%	62.50%	0.256
LIN28A	96	58.33%	70.00%	64.29%	65.12%	63.64%	0.285
QKI	100	78.00%	70.00%	74.00%	72.22%	76.09%	0.482
TARDBP	100	22.00%	70.00%	46.00%	42.31%	47.30%	–0.091
Weighted average		**64.41%**	**70.00%**	**67.25%**	**67.59%**	**66.94%**	**0.345**
DeepBind							
FUS	100	32.00%	33.00%	32.50%	32.32%	32.67%	–0.350
FXR1	97	32.99%	42.00%	37.56%	35.56%	39.25%	–0.251
FXR2	93	43.01%	73.00%	58.55%	59.70%	57.94%	0.168
IGF2BP2	94	48.94%	59.00%	54.12%	52.87%	55.14%	0.080
LIN28A	96	36.46%	53.00%	44.90%	42.68%	46.49%	-0.107
QKI	100	82.00%	81.00%	81.50%	81.19%	81.82%	0.630
TARDBP	100	50.00%	86.00%	68.00%	78.12%	63.24%	0.386
Weighted average		**46.62%**	**61.00%**	**53.91%**	**53.73%**	**54.05%**	**0.077**
catRAPID							
		DP value					
FUS	10	16.40%	–	–	–	–	–
FXR1	10	17.60%	–	–	–	–	–
FXR2	10	22.30%	–	–	–	–	–
IGF2BP2	10	16.70%	–	–	–	–	–
LIN28A	10	19.10%	–	–	–	–	–
QKI	10	15.50%	–	–	–	–	–
TARDBP	10	18.10%	–	–	–	–	–
Weighted average		**18.22%**	–	–	–	–	–

Sensitivity is shown for our model and DeepBind, and discriminative power (DP) value is shown for catRAPID. The specificity of our method is the same for all RBPs because it used a same set of negative data for all RBPs with a single model, whereas DeepBind has distinct models for each RBP. Due to the speed of the catRAPID server, catRAPID was tested on 10 RBP-binding sequences of 51 nucleotides for each RBP, whereas both our model and DeepBind were tested on all the RBP-binding sequences. Detailed results are available in Additional file 12

## Results and discussion

### Evaluation of feature contribution

Table 4 compares different combinations of features in 10-fold cross validation of our SVM model with the 1:1 training dataset. Among the single features, mPWM and dPWM were much better than nucleotide compositions. With mPWM or dPWM alone, the SVM model achieved an accuracy above 89% and an MCC above 0.79. This result indicates that mPWM and dPWM are very powerful features in predicting protein-binding regions in RNA sequences. Compared to using single features alone, using two different features resulted in performance improvement in sensitivity, accuracy, NPV and MCC. Nucleotide compositions alone achieved a much lower performance than sequence profiles of log-odds scores of mono-nucleotides and those of di-nucleotides, but performance gain was obtained with combination of nucleotide compositions and sequence profiles (sensitivity of 91.61%, specificity of 92.39%, accuracy of 92.02%, PPV of 91.69%, NPV of 92.31% and MCC of 0.840).

**Table 4:** Comparison of different combinations of features in 10-fold cross validation

	Sensitivity	Specificity	Accuracy	PPV	NPV	MCC
mPWM	89.09%	90.60%	89.87%	89.67%	90.06%	0.797
dPWM	90.48%	92.06%	91.31%	91.27%	91.34%	0.826
compositions	71.44%	88.23%	80.20%	84.76%	77.12%	0.608
mPWM + dPWM	91.46%	91.98%	91.73%	91.27%	92.16%	0.834
mPWM + compositions	91.31%	91.55%	91.43%	90.83%	92.00%	0.828
dPWM + compositions	91.07%	92.53%	91.83%	91.78%	91.88%	0.836
mPWM + dPWM + compositions	**91.61%**	**92.39%**	**92.02%**	**91.69%**	**92.31%**	**0.840**

Using all 3 features showed the best performance. mPWM: mono-nucleotide position weight matrix, dPWM: di-nucleotide position weight matrix, compositions: frequency of mono-nucleotides, di-nucleotides, and tri-nucleotides in the RNA sequence

### Cross validations

Table 5 shows the results of the standard 10-fold cross validations of the SVM model with the RBF kernel and random forest model with the 1:1, 1:2, 1:4, 1:6, 1:8 and 1:10 training datasets. The best performance of the SVM model observed in the balanced dataset with 1:1 ratio of positive to negative instances (sensitivity of 91.61%, specificity of 92.39%, accuracy of 92.02%, PPV of 91.69%, NPV of 92.31% and MCC of 0.840). As expected, running the SVM model on unbalanced datasets resulted in lower performances on average than running it on the balanced dataset with 1:1 ratio of positive to negative instances. In particular, PPV and MCC were significantly decreased as the ratio of negative instances was increased. But, NPV was rather increased slightly.

**Table 5:** Results of 10-fold cross validations of SVM and random forest on 6 datasets with different P:N ratios of positive to negative instances

P:N	Sensitivity	Specificity	Accuracy	PPV	NPV	MCC
SVM						
1:1	**91.61%**	92.39%	92.02%	91.69%	92.31%	0.840
1:2	**91.37%**	92.17%	91.91%	84.53%	95.80%	0.819
1:4	**91.13%**	92.33%	92.09%	74.64%	97.68%	0.777
1:6	**91.22%**	91.95%	91.84%	66.71%	98.34%	0.736
1:8	**91.22%**	91.92%	91.83%	62.52%	98.61%	0.713
1:10	**91.19%**	91.54%	91.50%	58.11%	98.78%	0.686
Random forest						
1:1	**91.13%**	92.06%	91.62%	91.32%	91.89%	0.832
1:2	**85.44%**	95.21%	92.09%	89.31%	93.32%	0.816
1:4	**80.40%**	97.18%	93.85%	87.59%	95.24%	0.802
1:6	**77.88%**	97.77%	94.78%	86.01%	96.16%	0.788
1:8	**76.01%**	98.01%	95.18%	84.95%	96.51%	0.777
1:10	**75.24%**	98.14%	95.53%	83.90%	96.86%	0.770

*PPV* positive prediction value, *NPV* negative prediction value, *MCC* Matthews correlation coefficient

As the dataset contains more negative instances, sensitivity, PPV and MCC of the random forest model were decreased. In particular, it showed a substantial decrease in sensitivity. Since there are much more non-binding sites than binding sites in actual RNA sequences, we determined that finding all possible binding sites at the expense of low PPV is better than missing the binding sites. Thus, we selected the SVM model as the final model for the web server.

As stated earlier, the SVM model with the RBF kernel is known to be better than the SVM with linear kernel when the number of instances is much larger than the number of features. For comparative purposes, we built an SVM model with linear kernel and performed 10-fold cross validation of the model (Additional file 6). The SVM model with linear kernel showed a slightly lower performance than the SVM model with the RBF kernel.

Our SVM model uses the protein sequence as an additional information when it is available. Additional file 7 shows the results of 10-fold cross validation of the SVM model when it is given a protein sequence in addition to an RNA sequence. The best performance was observed in the balanced dataset with 1:1 ratio of positive to negative instances (sensitivity of 93.18%, specificity of 92.01%, accuracy of 92.57%, PPV of 91.44%, NPV of 93.64% and MCC of 0.851).

Results of LOPO cross validation with respect to RBPs in the 1:1 training dataset are shown in Table 6. Since different RBPs have very different numbers of known RBP-binding regions, we examined a weighted average of performance measures instead of a simple average of them. The weighted average was computed from the total values of TP, FP, TN and FN of all runs. In LOPO cross validation, the model showed a sensitivity of 85.54%, a specificity of 89.53%, an accuracy of 87.60%, a PPV of 88.42%, an NPV of 86.89% and an MCC of 0.752. This result indicates that LOPO cross validation of our SVM model obtained a lower performance than 10-fold cross validation, but its average performance is reasonably high.

**Table 6:** Results of LOPO cross validation of our method with respect to 14 RBPs

	TP	TN	FP	FN	Sensitivity	Specificity	Accuracy	PPV	NPV	MCC
AGO1	37	50	3	18	67.27%	94.34%	80.56%	92.50%	73.53%	0.638
AGO2	39	49	2	18	68.42%	96.08%	81.48%	95.12%	73.13%	0.664
EWSR1	200	198	14	14	93.46%	93.40%	93.43%	93.46%	93.40%	0.869
FUS	468	534	46	19	96.10%	92.07%	93.91%	91.05%	96.56%	0.879
FXR1	3	7	0	1	75.00%	100.00%	90.91%	100.00%	87.50%	0.810
FXR2	25	33	1	11	69.44%	97.06%	82.86%	96.15%	75.00%	0.688
IGF2BP2	57	55	7	15	79.17%	88.71%	83.58%	89.06%	78.57%	0.678
LIN28A	221	263	25	57	79.50%	91.32%	85.51%	89.84%	82.19%	0.714
LIN28B	2214	2343	329	227	90.70%	87.69%	89.13%	87.06%	91.17%	0.783
QKI	3	5	0	1	75.00%	100.00%	88.89%	100.00%	83.33%	0.791
TAF15	11	16	1	2	84.62%	94.12%	90.00%	91.67%	88.89%	0.796
TARDBP	39	159	14	149	20.74%	91.91%	54.85%	73.58%	51.62%	0.179
YTHDF2	35	39	5	6	85.37%	88.64%	87.06%	87.50%	86.67%	0.741
ZC3H7B	388	438	43	94	80.50%	91.06%	85.77%	90.02%	82.33%	0.720
Total	3,740	4,189	490	632						
Weighted average					**85.54%**	**89.53%**	**87.60%**	**88.42%**	**86.89%**	**0.752**

The weighted average was computed from the total values of TP, TN, FP and FN of all runs. TP: true positive, *TN* true negative, *FP* false positive, *FN* false negative, *PPV* positive prediction value, *NPV* negative prediction value, *MCC* Matthews correlation coefficient

### Independent tests

For rigorous evaluation of our model, we tested it on independent datasets (30% of the entire data), which were not used in training the model. As in the 10-fold cross validation, we tested it on six test datasets with different ratios of positive to negative instances (called 1:1, 1:2, 1:4, 1:6, 1:8, and 1:10 test datasets hereafter). As shown in Table 7, the specificity, PPV and MCC were decreased as the ratio of negative instances was increased.

**Table 7:** Results of independent testing of our method on 6 datasets with different P:N ratios of positive to negative instances

P:N	Sensitivity	Specificity	Accuracy	PPV	NPV	MCC
1:1	**72.50%**	**91.90%**	**82.20%**	**89.95%**	**76.97%**	**0.656**
1:2	72.40%	91.80%	85.33%	**81.53%**	86.93%	**0.663**
1:4	74.10%	91.10%	87.70%	**67.55%**	83.36%	**0.630**
1:6	77.00%	90.26%	88.37%	**56.87%**	95.92%	**0.596**
1:8	77.80%	89.68%	88.36%	**48.53%**	97.00%	**0.554**
1:10	79.10%	89.70%	88.73%	**43.44%**	97.72%	**0.532**

*PPV* positive prediction value, *NPV* negative prediction value, *MCC* Matthews correlation coefficient

In particular, PPV and MCC were significantly decreased as the dataset contains more negative instances. This trend was also observed in 10-fold cross validation. However, other performance measures (sensitivity, accuracy, and NPV) were rather increased, and specificity was decreased slightly.

**Fig. 3:**
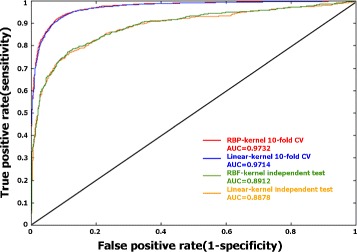
ROC curves of 10-fold cross validation and independent testing of the RBF-SVM and the linear SVM. Both in 10-fold cross validation and independent testing, the SVM model with the RBF kernel yielded a slightly larger area under the ROC curve (AUC) than the SVM model with linear kernel

Figure 3 shows the ROC curves of 10-fold cross validation and independent testing of the SVM models. In 10-fold cross validation, the SVM model with the RBF kernel yielded a slightly larger area under the ROC curve (AUC = 0.9732) than the SVM model with linear kernel (AUC = 0.9714). Likewise, in independent testing the SVM model with RBF kernel showed a slightly larger AUC (0.8912) than the SVM with linear kernel (0.8878).

Since the prediction model was trained with RBP-binding RNA sequences of 25 nucleotides, we examined whether it is applicable to RNAs of different sizes. For RNAs of *k* nucleotides (*k*<25), we extracted a total of 12,576 RBP-binding RNAs from CLIPdb. When testing the model on each RNA sequence with <25 nucleotides, we selected a position in the RNA sequence which results in the maximum sum of log-odds scores from an ungapped alignment of the sequence with mPWM. Based on the selected position, we encoded both mPWM and dPWM features and filled zeros for matrix elements that have no corresponding nucleotides in the RNA sequence to make the size of the feature vector comparable to those for 25-mer RNAs. Nucleotide compositions of short RNA sequences were encoded in the same way as RNA sequences of 25 nucleotides. The prediction performance with short RNA sequences was lower than that with 25-mer RNAs, but its accuracy is as high as 74.4% (Additional file 8). We also tested the prediction model on RNA sequences with >25 nucleotides, and details are discussed in the next section. Additional file 9 shows the change in accuracy of the model for RNA sequences with lengths between 21 and 40 nucleotides.

Without changing the original mPWM and dPWM, we tested our model for new RBPs that were not considered in constructing datasets. It showed a low performance for some RBPs but obtained a high performance for some RBPs (Additional file 10). The best performance was observed for HNRNPD (sensitivity of 94.29%, specificity of 94.37%, accuracy of 94.33%, PPV of 92.52%, NPV of 95.71% and MCC of 0.884).

A negative dataset in our study was constructed by random selection. For comparative purposes, we constructed different negative datasets by extracting a subsequence in the upstream region of each RBP binding region. We tried several different distances ranging from 1 to 1001 nucleotides between the negative instance and the positive instance (i.e., RBP binding region) in a same RNA sequence. The performance of our model with a new negative dataset was as high as that with the previous dataset in which negative instances were sampled randomly. The specificity has been increased slightly with the new negative dataset. Details are available in Additional file 11.

### Comparison with other methods

For the comparison with DeepBind and catRAPID, we prepared two new datasets of RBP-binding RNA sequences. The first test dataset consists of RNA sequences of 25 nucleotides extracted from CLIPdb. In the first dataset, similar sequences with any in the training dataset were removed by running CD-HIT-EST with a cut-off value of 0.8. The second test dataset was constructed by adding 13 nucleotides in the original genome sequence at both ends of the 25-mer RNAs in the first dataset. The reason that we could not use RBP-binding RNA sequences of 51 nucleotides in CLIPdb is because DeepBind does not provide a prediction model for RBP-binding RNA sequences of 51 nucleotides (DeepBind provides distinct models for each RBP). For negative data of the test datasets, we selected 100 non-binding regions of 25 and 51 nucleotides in the reference human genome GRCh37/hg19.

When testing the model on each RNA sequence with >25 nucleotides, we found a 25-mer subsequence of the RNA sequence which results in the maximum sum of log-odds scores from an alignment of the 25-mer subsequence with mPWM. In a feature vector, we encoded both mPWM and dPWM features of the selected 25-mer subsequence along with nucleotide compositions of the entire RNA sequence.

Table 2 shows the results of testing our model and DeepBind on RBP-binding sequences for 7 RBPs. In predicting RBP-binding regions of 25 nucleotides, our model achieved an average sensitivity of 70.72%, specificity of 94.00%, accuracy of 83.83%, PPV of 90.14%, NPV of 80.54% and MCC of 0.676. DeepBind showed very low scores for most RBP-binding sequences, but the scores of DeepBind are known to be on an arbitrary scale [9]. Thus, for a fair comparison, we computed Z-scores of DeepBind scores. If an RNA sequence tested by DeepBind had a Z-score >0, it was considered as RBP-binding; otherwise, it was considered as non-binding. DeepBind showed an average sensitivity of 36.28%, specificity of 58.71%, accuracy of 48.91%, PPV of 40.53%, NPV of 54.29% and MCC of -0.051, which is much lower than ours.

In testing on RBP-binding regions of 51 nucleotides, our model showed a much better performance than DeepBind (Table 3). Our model obtained an average sensitivity of 64.41%, specificity of 70.00%, accuracy of 67.25%, PPV of 67.59%, NPV of 66.94% and MCC of 0.345, whereas DeepBind showed an average sensitivity of 46.42%, specificity of 61.00%, accuracy of 53.91%, PPV of 53.73%, NPV of 54.05% and MCC of 0.077. The catRAPID server was too slow to test all RBP-binding sequences shown in Table 3, so it was tested on 10 RBP-binding sequences for each RBP. catRAPID showed low discriminative power (DP) values in most test cases. Since DP of catRAPID represents the interaction propensity of a protein—RNA pair with respect to the training sets [8], the result of testing catRAPID on RBP-binding sequences indicates a low confidence level of the prediction. Details of the RBP-binding sequences used for comparison of three methods and raw data obtained from execution of the three methods are available in Additional file 12.

## Conclusion

In this paper we proposed a new computational method to predict protein-binding regions in mRNA sequences using sequence profiles constructed from log-odds scores of mono- and di-nucleotides and nucleotide compositions. The method has been implemented in SVM models and evaluated in several ways, including standard 10-fold cross validation on six datasets with different ratios of positive to negative instances, LOPO cross validation, and independent testing with six datasets of different ratios of positive to negative instances. We also compared our method with DeepBind and catRAPID using another test dataset.

Results of cross validation and independent testing of the method on actual RBP-binding regions in human mRNAs showed that sequence profiles of log-odds scores of mono- and di-nucleotides are much more powerful features than nucleotide compositions in finding protein-binding regions in RNA sequences. Nucleotide compositions alone achieved a much lower performance than sequence profiles of log-odds scores of mono-nucleotides and those of di-nucleotides, but performance gain was obtained with combination of nucleotide compositions and sequence profiles. The best performance was observed in a balanced dataset of positive and negative instances. 10-fold cross validation with a balanced dataset achieved a sensitivity of 91.6%, a specificity of 92.4%, an accuracy of 92.0%, a PPV of 91.7%, an NPV of 92.3% and an MCC of 0.84. 10-fold cross validation of RNA and protein sequence feature vector model with a balanced dataset achieved a sensitivity of 93.2%, a specificity of 92.0%, an accuracy of 92.6%, a PPV of 91.4%, an NPV of 93.6% and an MCC of 0.85. LOPO cross validation showed a lower performance than the 10-fold cross validation, but the performance remains high (sensitivity of 85.5%, specificity of 89.5%, accuracy of 87.6%, PPV of 88.4%, NPV of 86.9% and MCC of 0.752). In testing the model on independent datasets, it achieved a sensitivity of 72.5%, a specificity of 91.9%, an accuracy of 82.2%, a PPV of 89.9%, an NPV of 77.0% and an MCC of 0.66. Testing of our model and two other methods showed that our model is better than the others.

The results shown in this paper are preliminary, but demonstrate the potential of our method to predict RBP-binding regions in mRNA. Given that the average length of human mRNAs is about 2 kb and that different RBPs have different binding preferences within an mRNA, it is not straightforward to find RBP binding regions in mRNAs. A computational method like ours will help biologists save time and effort in designing and performing their in vivo or in vitro experiments to detect protein-RNA binding sites by narrowing down candidate binding regions on target RNAs.

## Additional files

Additional file 1 Type of RBP binding regions. Type of RBP binding regions in human mRNAs. (ZIP 429 kb)

Additional file 2 Histogram of the length of RBP-binding regions in CLIPdb. Distribution of the length of RNA sequences binding with 14 RBPs. nt: length in nucleotides of the RBP-binding regions. (PNG 22 kb)

Additional file 3 5,145 RBP-binding regions. 5,145 RBP-binding regions in human mRNA sequences obtained from CLIPdb. For each binding region, RBP name, chromosome name, the starting position of the binding region in the chromosome, the ending position of the binding region in the chromosome, binding affinity score, and strand information are specified. (XLSX 249 kb)

Additional file 4 6 training datasets with different ratios of positive to negative instances. 6 training datasets with different ratios of positive to negative instances (called 1:1, 1:2, 1:4, 1:6, 1:8 and 1:10 training datasets). (XLSX 3553 kb)

Additional file 5 6 test datasets with different ratios of positive to negative instances. 6 test datasets with different ratios of positive to negative instances (called 1:1, 1:2, 1:4, 1:6, 1:8 and 1:10 test datasets). (XLSX 1208 kb)

Additional file 6 Results of 10-fold cross validation of the SVM model with linear kernel with 6 train datasets. The performance of the SVM model with linear kernel with different ratios of positive to negative instances. (DOCX 17 kb)

Additional file 7 Results of 10-fold cross validation of the SVM model using both RNA and protein features. The performance of the SVM model that uses protein features as well as RNA features in 6 different datasets. (DOCX 17 kb)

Additional file 8 Results of testing our model on RNA sequences shorter than 25 nucleotides. The performance of the SVM model with RNA sequences shorter than 25 nucleotides. (DOCX 18 kb)

Additional file 9 Results of testing our model on RNA sequences with length between 21 and 40 nucleotides. (PNG 50 kb)

Additional file 10 Results of testing our model for new RBPs. Results of testing our model on predicting RBP binding regions in RNA for new RBPs. (DOCX 18 kb)

Additional file 11 Results of testing our model on RNA sequences with different negative datasets. The performance of our model with different negative datasets whose instances were selected in the upstream region of each RBP binding region. (XLSX 464 kb)

Additional file 12 Results of testing DeepBind and catRAPID on RNA sequences of 25 and 51 nucleotides. RBP-binding sequences used for comparison of DeepBind and catRAPID prediction methods and raw data obtained from execution of the three methods. (ZIP 223 kb)

## Abbreviations

CDS: Coding sequence
CLIP: Cross-linking and immunoprecipitation
dC: Di-nucleotide composition
dPWM: Di-nucleotide position weight matrix
FN: False negative
FP: False positive
LIBSVM: Library for support vector machine
LOPO: Leave-one-protein-out
mC: Mono-nucleotide composition
MCC: Matthews correlation coefficient
mPWM: Mono-nucleotide position weight matrix
NPV: Negitive predictive value
PPI: Protrin-protein interaction
PPV: Positive predictive value
PWM: Position weight matrix
RBF: Radial basis function
RBP: RNA-binding protein
SVM: Support vector machine
tC: Tri-nucleotide composition
TN: True negative
TP: True positive
