# Supplementary figures and images for: Predicting protein-binding regions in RNA using nucleotide profiles and compositions
Source: BMC Syst Biol. 2017 Mar 14;11(Suppl 2):16. doi: 10.1186/s12918-017-0386-4 (PMC5374631; doi:10.1186/s12918-017-0386-4)

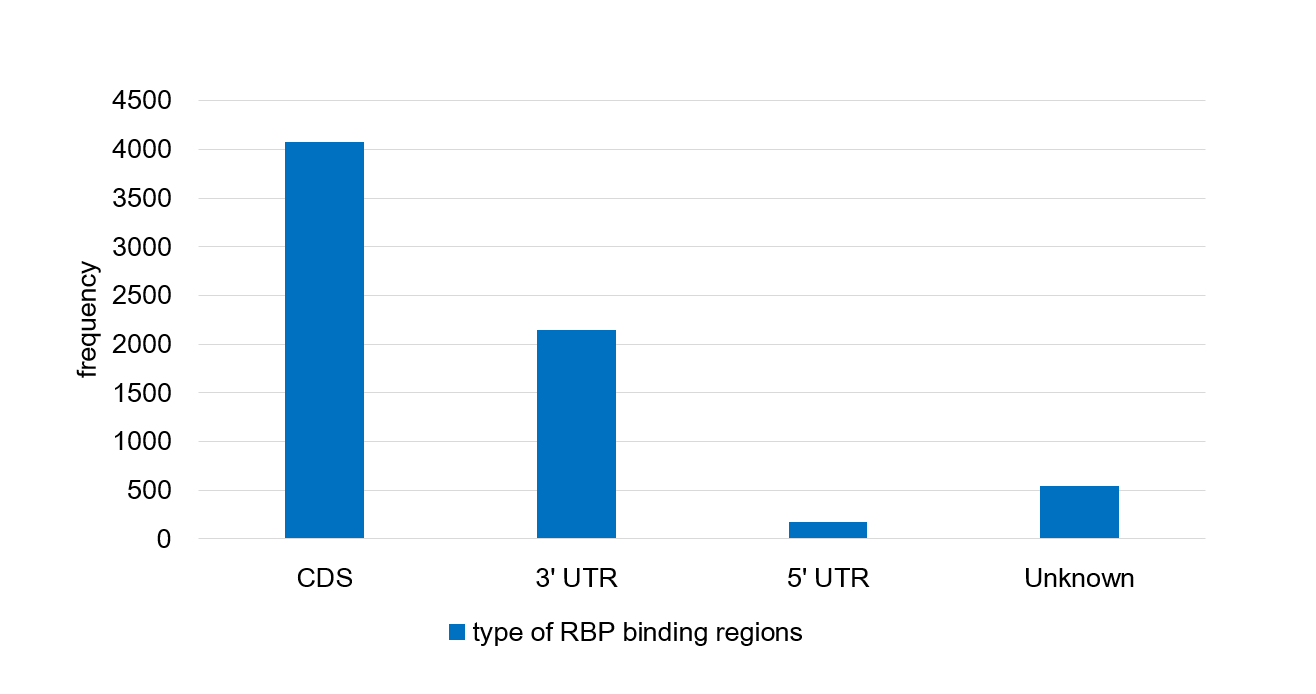

Supplement: Supplementary file 1 — Type of RBP binding regions. Type of RBP binding regions in human mRNAs. (ZIP 429 kb) [file 12918_2017_386_MOESM1_ESM.zip › Additional_file_1.png]

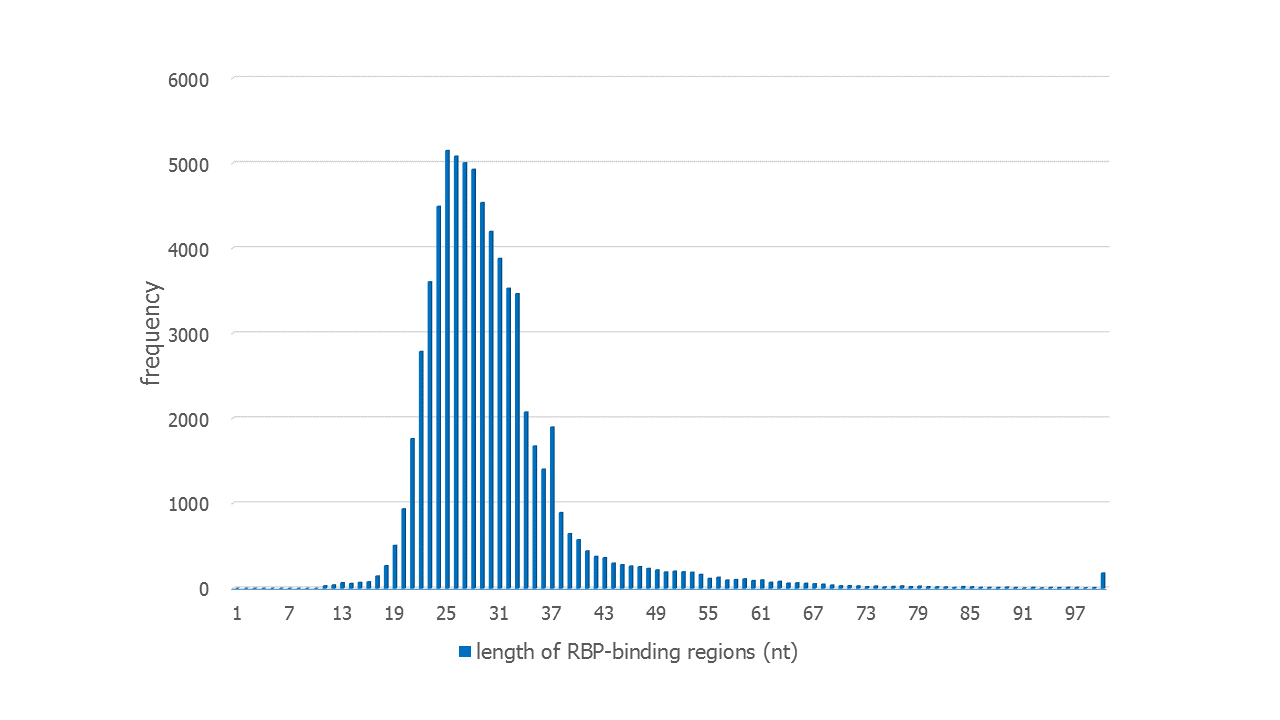

Supplement: Supplementary file 2 — Histogram of the length of RBP-binding regions in CLIPdb. Distribution of the length of RNA sequences binding with 14 RBPs. nt: length in nucleotides of the RBP-binding regions. (PNG 22 kb) [file 12918_2017_386_MOESM2_ESM.png]

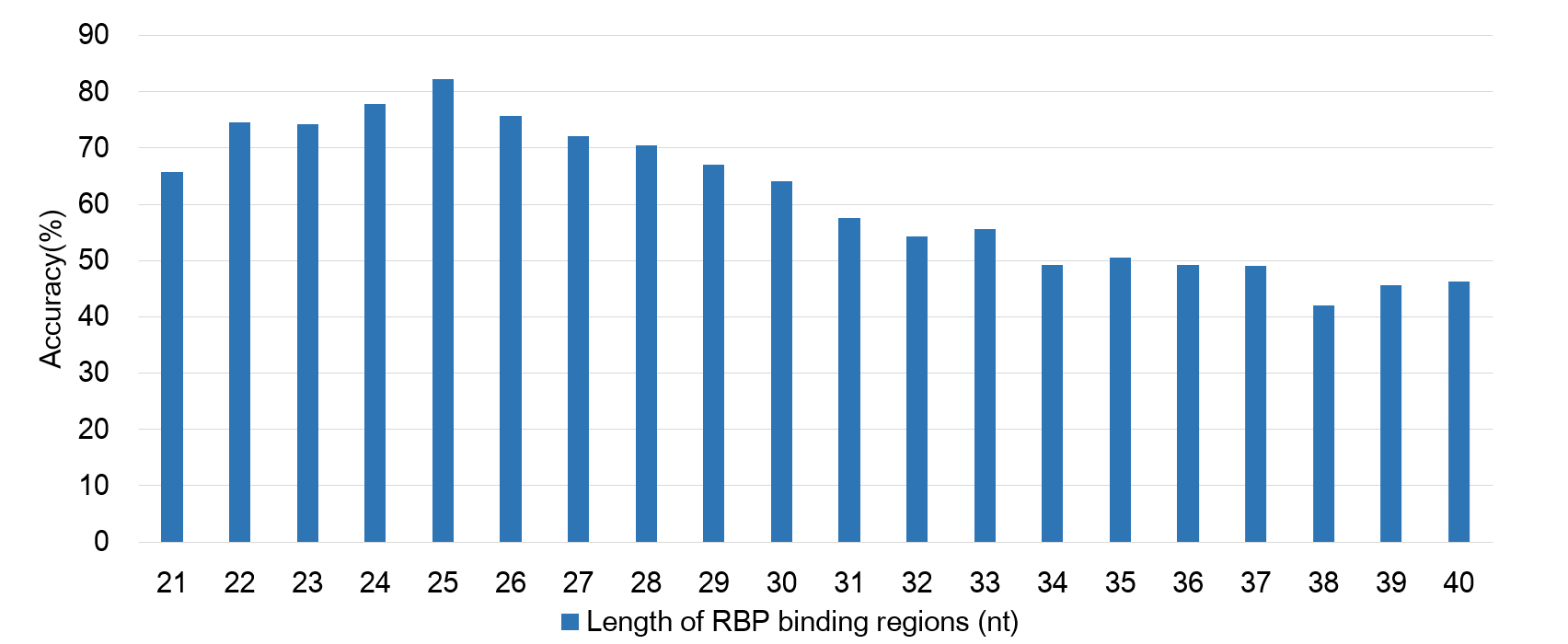

Supplement: Supplementary file 9 — Results of testing our model on RNA sequences with length between 21 and 40 nucleotides. (PNG 50 kb) [file 12918_2017_386_MOESM9_ESM.png]
