# Supplementary material for: Predicting protein-binding regions in RNA using nucleotide profiles and compositions
Source: BMC Syst Biol. 2017 Mar 14;11(Suppl 2):16. doi: 10.1186/s12918-017-0386-4 (PMC5374631; doi:10.1186/s12918-017-0386-4)
Supplement: Supplementary file 6 — Results of 10-fold cross validation of the SVM model with linear kernel with 6 train datasets. The performance of the SVM model with linear kernel with different ratios of positive to negative instances. (DOCX 17 kb) [file 12918_2017_386_MOESM6_ESM.docx]

Additional file 6 – Results of 10-fold cross validation of the SVM model with linear kernel model with 6 train datasets.

| P:N | sensitivity(%) | specificity(%) | accuracy(%) | PPV(%) | NPV(%) | MCC | AUC |
| --- | --- | --- | --- | --- | --- | --- | --- |
| 1:1 | 91.34 | 92.09 | 91.73 | 91.37 | 92.07 | 0.834 | 0.971602 |
| 1:2 | 91.49 | 91.43 | 91.45 | 83.33 | 95.82 | 0.810 | 0.969658 |
| 1:4 | 91.90 | 91.34 | 91.46 | 72.46 | 97.85 | 0.765 | 0.970653 |
| 1:6 | 92.02 | 90.79 | 90.97 | 63.86 | 98.47 | 0.718 | 0.969102 |
| 1:8 | 92.73 | 90.23 | 90.55 | 58.37 | 98.82 | 0.689 | 0.968311 |
| 1:10 | 92.85 | 89.80 | 90.15 | 53.95 | 98.98 | 0.662 | 0.968171 |
