# Supplementary material for: Predicting protein-binding regions in RNA using nucleotide profiles and compositions
Source: BMC Syst Biol. 2017 Mar 14;11(Suppl 2):16. doi: 10.1186/s12918-017-0386-4 (PMC5374631; doi:10.1186/s12918-017-0386-4)
Supplement: Supplementary file 7 — Results of 10-fold cross validation of the SVM model using both RNA and protein features. The performance of the SVM model that uses protein features as well as RNA features in 6 different datasets. (DOCX 17 kb) [file 12918_2017_386_MOESM7_ESM.docx]

Additional file 7 - Results of 10-fold cross validation of an SVM model that uses protein features as well as RNA features. Cross validation was conducted using 6 datasets with different ratios of positive to negative instances.

| P:N | sensitivity (%) | specificity (%) | accuracy (%) | PPV (%) | NPV (%) | MCC | AUC |
| --- | --- | --- | --- | --- | --- | --- | --- |
| 1:1 | 93.18 | 92.01 | 92.57 | 91.44 | 93.64 | 0.851 | 0.977919 |
| 1:2 | 93.86 | 92.76 | 93.11 | 85.87 | 96.99 | 0.847 | 0.981703 |
| 1:4 | 93.33 | 93.44 | 93.42 | 77.90 | 98.26 | 0.813 | 0.981852 |
| 1:6 | 93.45 | 93.53 | 93.52 | 71.86 | 98.78 | 0.784 | 0.980782 |
| 1:8 | 92.82 | 93.28 | 93.22 | 67.10 | 98.88 | 0.754 | 0.978744 |
| 1:10 | 92.91 | 93.08 | 93.06 | 63.34 | 99.03 | 0.732 | 0.978239 |
