# Supplementary material for: Predicting protein-binding regions in RNA using nucleotide profiles and compositions
Source: BMC Syst Biol. 2017 Mar 14;11(Suppl 2):16. doi: 10.1186/s12918-017-0386-4 (PMC5374631; doi:10.1186/s12918-017-0386-4)
Supplement: Supplementary file 8 — Results of testing our model on RNA sequences shorter than 25 nucleotides. The performance of the SVM model with RNA sequences shorter than 25 nucleotides. (DOCX 18 kb) [file 12918_2017_386_MOESM8_ESM.docx]

Additional file 8 – Results of testing our model on RNA sequences shorter than 25 nucleotides.

| length of the RBP-binding regions | #RBP-binding regions | sensitivity (%) | specificity (%) | accuracy (%) | PPV (%) | NPV(%) | MCC |
| --- | --- | --- | --- | --- | --- | --- | --- |
| 21 | 1,747 | 92.50 | 38.81 | 65.66 | 60.19 | 83.81 | 0.371 |
| 22 | 2,771 | 93.87 | 55.13 | 74.50 | 67.66 | 89.98 | 0.531 |
| 23 | 3,588 | 90.67 | 57.64 | 74.16 | 68.17 | 86.06 | 0.512 |
| 24 | 4,470 | 82.66 | 73.06 | 77.86 | 75.42 | 80.82 | 0.560 |
| total | 12,576 | - | - | - | - | - | - |
| weighted average | - | 88.78 | 59.95 | 74.37 | 68.92 | 84.23 | 0.509 |
