# Supplementary material for: Predicting protein-binding regions in RNA using nucleotide profiles and compositions
Source: BMC Syst Biol. 2017 Mar 14;11(Suppl 2):16. doi: 10.1186/s12918-017-0386-4 (PMC5374631; doi:10.1186/s12918-017-0386-4)
Supplement: Supplementary file 10 — Results of testing our model for new RBPs. Results of testing our model on predicting RBP binding regions in RNA for new RBPs. (DOCX 18 kb) [file 12918_2017_386_MOESM10_ESM.docx]

Additional file 10 – Results of predicting RBP binding regions in RNA for new RBPs.

| RBP | #RBP-binding regions in RNA | sensitivity (%) | specificity (%) | accuracy (%) | PPV (%) | NPV (%) | MCC |
| --- | --- | --- | --- | --- | --- | --- | --- |
| AGO3 | 84 | 57.14 | 89.29 | 73.21 | 84.21 | 67.57 | 0.490 |
| CARPRIN1 | 980 | 85.32 | 91.02 | 88.25 | 89.97 | 86.78 | 0.765 |
| CPSF3 | 180 | 50.59 | 89.94 | 70.21 | 83.50 | 64.41 | 0.441 |
| CPSF2 | 76 | 75.81 | 91.67 | 84.33 | 88.68 | 81.48 | 0.688 |
| CPSF4 | 103 | 67.71 | 92.55 | 80.00 | 90.28 | 73.73 | 0.621 |
| ELAVL1 | 526 | 66.67 | 100.00 | 83.33 | 100.00 | 75.00 | 0.707 |
| HNRNPD | 160 | 94.29 | 94.37 | 94.33 | 92.52 | 95.71 | 0.884 |
| IGF2BP1 | 2,105 | 57.33 | 89.33 | 78.67 | 72.88 | 80.72 | 0.500 |
| IGF2BP3 | 351 | 61.04 | 89.61 | 80.09 | 74.60 | 82.14 | 0.536 |
| MOV10 | 926 | 86.92 | 90.97 | 89.04 | 89.74 | 88.44 | 0.780 |
